# Special Interest to the Dental Profession

**Published:** 1899-10

**Authors:** 


### EDITORIAL.

### Special Interest to the Dental Profession.

The following statement has just come to hand: "Acting on the recent decision of the U. S. Circuit Court for the Southern District of New York, establishing as it is claimed the validity of the patent issued in 1881 to James E. Low for crown and bridge dental work, the present holders of the patent have placed attachments on the offices of seven Boston dentists. The patent has been the cause of an immense amount of litigation, and the end is not yet. After the New York decision a circular was sent to all dentists, requesting that they pay $25 a year for each year during which they have used the work. Some of them it is said have agreed to this demand, but many of them are determined to avoid payment if possible. They have organized to fight the case. They say that the matter will be taken to the U. S. Court of Appeals and the U. S. Supreme Court if necessary."

All dentists are doubtless aware that the Dental Protective Association of the United States has been in operation for many years. The object of which is the protection of its members, from the operation of any invalid patents, pertaining to dental practice or work of any sort.

In 1888 the Crown & Bridge Co. secured a decision by the highest court in the Southern District of New York, declaring some of the various patents on crown and bridge work valid. With this in their favor, the Crown & Bridge Co. immediately started through the country collecting royalty, terms imposed about $25 per year license fee, and 15 percent on all work done. And not only this, but those dentists who yielded to these demands signed a license and consented thereby to the validity of more than thirty different patents which the Crown Co. had secured on various devices.

Soon after this the Protective Association was organized, and within six weeks it had stopped the Crown Co. from enforcing their claims, driving them from one court to another, they withdrawing and paying costs rather than to make a test case, until they reached the Federal district in which they had previously obtained their favorable decision, here the Protective Association had the former decision of these courts reversed and the Low bridge patent declared invalid, first before the judge of the Circuit Court and afterward in the U. S. Court of Appeals. This it was presumed would end the fighting with the Crown Co.

This condition of things was the occasion of the conclusion, in the minds of the profession, and especially to those who had not become members, that it was no longer necessary to become members of the Protective Association. This, however, as things have recently occurred, show that the conclusion was not well grounded.

Membership in this Association is the only practical means of escaping from the unjust demands that are so frequently made upon unjust and invalid patent claims. No member of the Dental Protective Association has ever thus far, as we are aware, been prosecuted or even threatened with prosecution, nor made to pay royalty by the Tooth Crown Co.

The Protective Association proposes and thus far has, and doubtless will in the future, protect all its members from loss in consequence of unrighteous demands based upon invalid patents. This being the fact, it is rather surprising that all persons in the profession using the work for which these claims are made, do not at once become members of the Dental Protective Association. It is the only way so far as now appears of avoiding the annoyance occasioned by such demands and by unjust prosecution. No dentist can alone, successfully resist, at least so far as cost and annoyance is concerned, the demands of the Tooth Crown Co.

So we have no hesitancy in recommending to every dentist, who is not in the city of refuge to flee into it, with all possible haste. The door is now open, how long it may be so is uncertain, as those already in begin to think it is about time they should not continue to pay the expense of litigation, the fruits of which result in benefit to the whole profession.

This Association in no case resists the legal rights of anybody, but unjust, oppressive and illegal demands upon the rights of others.

Now, it is to be hoped that every member of the profession not a member of the Protective Association will at once become so, by applying to Dr. J. N. Crouse, of Chicago, Chairman of the Dental Protective Association. He will be found, for the present at least, with the gate open for the entrance of all who desire to escape from an unjust prosecution.

